# Association of ADAM33 gene polymorphisms with COPD in a northeastern Chinese population

**DOI:** 10.1186/1471-2350-10-132

**Published:** 2009-12-10

**Authors:** Xinyan Wang, Lei Li, Jinling Xiao, Chengzhen Jin, Kun Huang, Xiaowen Kang, Xiaomei Wu, Fuzhen Lv

**Affiliations:** 1 Department of Respiratory, the Second Affiliated Hospital of Harbin Medical University, Harbin, 150081, PR China; 2 Department of Neurology, the Second Affiliated Hospital of Harbin Medical University, Harbin, 150081, PR China

## Abstract

**Background:**

Chronic obstructive pulmonary disease (COPD) is influenced by both environmental and genetic factors. ADAM33 (a disintegrin and metalloproteinase 33) has been one of the most exciting candidate genes for asthma since its first association with the disease in Caucasian populations. Recently, ADAM33 was shown to be associated with excessive decline of lung function and COPD. The aim of this study was to evaluate the potential relationship between polymorphisms of ADAM33 and COPD in a Han population in northeastern China.

**Methods:**

A total of 312 COPD patients and a control group of 319 healthy volunteers were recruited for this study. Eight polymorphic loci (V4, T+1, T2, T1, S2, S1, Q-1, and F+1) of ADAM33 were selected for genotyping. Genotypes were determined by using the polymerase chain reaction-restriction fragment length polymorphism (PCR-RFLP) method.

**Results:**

Statistically significant differences in the distributions of the T2G, T1G, S2C, and Q-1G alleles between patients and controls were observed (*P *< 0.001, odds ratio (OR) = 2.81, 95% confidence interval (CI) = 2.19-3.61; *P *< 0.001, OR = 2.60, 95% CI = 2.06-3.30; *P *= 0.03, OR = 1.31, 95% CI = 1.02-1.69; and *P *< 0.001, OR = 1.93, 95% CI = 1.50-2.50, respectively). Haplotype analysis showed that the frequencies of the CGGGGAGC, CGGGGAGT, CGGGCAGC, and CGGGGGGC haplotypes were significantly higher in the case group than in the control group (*P *= 0.0002, 0.0001, 0.0005, and 0.0074, respectively). In contrast, the haplotype CGAAGAGC was more common in the control group than in the case group (*P *< 0.0001).

**Conclusion:**

These preliminary results suggest an association between ADAM33 polymorphisms and COPD in a Chinese Han population.

## Background

Chronic obstructive pulmonary disease (COPD) is a major cause of chronic morbidity and mortality. The World Health Organization (WHO) listed COPD as the fifth leading cause of death in the world, and it is estimated that COPD will be the third most common cause of death globally by 2020 [1]. It is also an increasingly common problem in China. A survey of 20,245 participants in seven regions in China in 2007 indicated that the prevalence of COPD in adults aged ≥40 years was 8.2%. The disease is characterized by airflow limitation and is associated with an abnormal inflammatory response of the lungs in response to noxious particles or gases, especially cigarette smoke [1]. These products lead to emphysema and airway remodeling, which is manifested by squamous and mucous metaplasia of the epithelium, smooth muscle hypertrophy, and airway wall fibrosis. These pathological abnormalities interact synergistically to cause progressive airflow obstruction [2]. Cigarette smoke is by far the most important risk factor for COPD, but there is a wide range in disease severities, irrespective of the number of pack years of smoking. Furthermore, only a minority (10-20%) of smokers develop clinically significant COPD. This suggests that, aside from smoking, there are underlying genetic factors that play a role in the development and severity of COPD. Based on family studies, the estimated relative risk for genetic susceptibility to COPD is about three [3].

ADAM33, is a member of the ADAM (a disintegrin and metalloprotease) family. ADAM proteins are involved in cell adhesion, cell fusion, cell signaling, and proteolysis [4,5]. The latter can be illustrated by the capacity to shed cytokines, growth factors, or their receptors from the cell surface and the remodeling of extracellular matrix components. Garlisi demonstrated that ADAM33 is an active proteinase that is able to cleave α2-macroglobulin [6] and synthetic peptides [7]. The enzymatic activity of ADAM33 can be inhibited by tissue inhibitor of metalloproteinase-3 and -4 (TIMP-3 and -4, respectively) as well as several small molecules [7]. This suggests that ADAM33 is involved in pulmonary defenses and tissue remodeling. Chronic respiratory diseases such as COPD and asthma are characterized by airflow obstruction and a chronic, persistent inflammatory process. The inflammatory process is a complex interaction between many cellular mechanisms, inflammatory mediators, and their effects [8]. A crucial pathological feature of COPD is airway inflammation and remodeling. Puxeddu et al. [9] showed that a truncated, soluble form of ADAM33 containing the catalytic domain caused rapid induction of endothelial cell differentiation in vitro and angiogenesis ex vivo and in vivo. Although not as well studied, there is evidence that the vascular area of the airway is significantly increased in COPD and that this increase correlates with the degree of airflow obstruction [10].

Genome-wide screening revealed that chromosome 20p13 was significantly linked to asthma and airway hyperresponsiveness in 460 families with asthma from the UK and the USA. This genomic region contains the gene ADAM33 [11]. Since the first report of an association between ADAM33 polymorphisms and asthma in two Caucasian populations from the UK and the USA, a number of replication studies have been published with differing results [12-15]. The differences in the association results may be due to phenotypic and environmental heterogeneity between cohorts. Additional studies demonstrated that SNPs within the ADAM33 locus are associated with accelerated decline of lung function in the general population and in asthma patients [16,17]. The ADAM33 gene is expressed in airway smooth muscle cells and fibroblasts in the lung [18], suggesting that it is not only important in the development of asthma but also in disease progression, possibly through airway remodeling [19,20]. These latter findings suggest a function of ADAM33 related to lung growth and repair in general rather than solely associated with asthma. Recent studies revealed that SNPs within ADAM33 confer susceptibility to COPD in the general population and are associated with airway inflammation in COPD [21,22]. The aim of the current study was to determine whether ADAM33 SNPs are associated with COPD in a Chinese Han population.

## Methods

### Population subjects

COPD patients were recruited from the Second Affiliated Hospital of Harbin Medical University in China. A total of 312 patients with stable COPD who had not been previously treated with theophylline, β2-adrenergic receptor agonists, or glucocorticosteroids were recruited for this study. The diagnosis of COPD was established using the following criteria: (1) history of cigarette smoking (a minimum of 10 pack years) in patients who were current smokers at the time of evaluation; (2) no exposure (occupational or otherwise) to other substances known to cause lung disorders; (3) absence of atopy; (4) no history of systemic or other pulmonary disease or congenital and/or acquired systemic immunodeficiency; (5) forced expiratory volume in the first second (FEV1)/forced vital capacity (FVC) <70% and FEV1 after inhalation of 200 mg salbutamol <80%. The control group consisted of 319 unrelated subjects with healthy pulmonary function and no known medical illnesses or family disorders. Control subjects were patients visiting the same hospital for a health check-up or community volunteers. They were matched for age, gender, and smoking history with the case group. The characteristics of the study population are shown in Table 1. Participants were of Han origin and lived in Harbin, which is in northeast China.

**Table 1:** Description of the studied population

Characteristic	CASE	CONTROL
Number of patients	312	319
Age (years)	60.5 (7.8)	61.5 (8.1)
Male: Female	186:126	192:127
Pack years of smoking	35.46 (15.9)	32.54 (12.7)
FEV1 (% predicted)	52.5 (8.6)	91.5 (9.6)
FEV1/FVC (%)	47.5 (7.6)	90.5 (11.6)
Postbd FEV1 (% predicted)	58.5 (9.6)	93.5 (10.5)
Postbd FEV1/FVC (%)	49.2 (8.5)	91.5 (11.8)

Data are means and standard deviations (in parentheses)

Pack year: (packs per day) × (years smoked)

FEV1: forced expiratory volume in first second

FVC: forced vital capacity

Postbd: post bronchodilator

Approval from the local regional ethics committee was obtained before initiating the study. COPD patients and healthy controls were informed of the study protocol and provided written consent. Genomic DNA was extracted using a DNA Extraction Kit (Qiagen, Germany) from peripheral blood that was anticoagulated in ACD (acid-citrate dextrose).

### Genotyping by PCR-RFLP

Eight SNPs were chosen from published ADAM33 SNPs associated with excess decline in FEV1 and/or presence of COPD. Information on the selected SNPs is shown in Table 2. Genotyping was performed by polymerase chain reaction-restriction fragment length polymorphism (PCR-RFLP) analysis. The polymorphic region was amplified by PCR using a T-Gradient Thermoblock PCR System (Iometra, Germany) in a 25 μl reaction solution containing 0.3 μg genomic DNA, 10× PCR buffer, 0.3 mM MgCl_2_, 0.2 mM dNTPs, 2 U TaqDNA polymerase (Takara, Japan), and 0.1 μmol of each primer (Invitrogen, USA). Genotyping primers are shown in Table 2. PCR products were digested overnight with restriction enzymes (NEB, UK) according to the manufacturer's protocol and analyzed by agarose gel electrophoresis. The restriction enzymes and the length of the digested fragments are shown in Table 3.

**Table 2:** Description of the investigated ADAM33 SNPs

Chromosome Position	Reference SNP ID	SNP Name	Alleles	Primer Sequences
3589161	2787094	V4	C/G	F: 5'-ACACACAGAATGGGGGAGAG-3' R: 5'-CCAGAAGCAAAGGTCACACA-3'
3590127	2280089	T+1	A/G	F: 5'-CTGAGCCCAGAAACCTGATT-3' R: 5'-AGAAGGGAAGGGCTCATGC-3'
3590205	2280090	T2	A/G	F: 5'-TTCTCAGGGTCTGGGAGAAA-3' R: 5'-GCCAACCTCCTGGACTCTTA-3'
3590234	2280091	T1	A/G	F: 5'-ACTCAAGGTGACTGGGTGCT-3' R: 5'-GAGGGCATGAGGCTCACTTG-3'
3591742	528557	S2	C/G	F: 5'-AGAGCTCTGAGGAGGGGAAC-3' R: 5'-TGTGCAGGCTGAAAGTATGC-3'
3591765	3918396	S1	A/G	F: 5'-TGTGCAGGCTGAAAGTATGC-3' R: 5'-AGAGCTCTGAGGAGGGGAAC-3'
3592207	612709	Q-1	A/G	F: 5'-GGATTCAAACGGCAAGGAG-3' R: 5'-GTTCACCTAGATGGCCAGGA-3'
3595085	511898	F+1	C/T	F: 5'-CTGCCACAATGTACAGTTCCA-3' R: 5'-AAGGACTTCTCAACCCACGA-3'

**Table 3:** Restriction enzymes and lengths of digested fragments

	V4	T+1	T2	T1	S2	S1	Q-1	F+1
Enzyme	PstI	HpyAV	HpyCH4III	NcoI	FseI	HinfI	BtsCI	BsmBI
Length of digested fragments(bp)	G:	A:	A:	A:	C:	G:	A:	C:
	168+206	284+28	198+112	140+260	148+156	132+172	20+138	208+192
	C:374	G:312	G:310	G:400	G:304	A:304	G:158	T:400

### Statistical analyses

Each SNP was tested for deviation from Hardy-Weinberg equilibrium (HWE) using the χ^2 ^statistic with expected frequencies derived from allele frequencies. SNPs were excluded from the analysis if they were out of HWE (*P *< 0.05). The allele frequencies for the case group and control group individuals were statistically compared using the χ^2 ^test with SPSS software for Windows (version 14.0; SPSS Inc, Chicago, IL). Haplotype frequencies for multiple sites in phase-unknown cases and controls were estimated using the expectation-maximization method of Haploview 4.1 software http://www.broad.mit.edu/mpg/haploview/. The relative risk associated with rare alleles was estimated as an odds ratio (OR) with a 95% confidence interval (CI).

## Results

### Distribution of eight polymorphisms in the case and control groups

Deviations from Hardy-Weinberg equilibrium were not seen in the COPD or control group for any polymorphisms. The genotype frequencies of the ADAM33 SNPS in the COPD and control groups are shown in Table 4. The genotype and allele frequencies were compared between the two groups. The frequencies of the T2G allele, the T1G allele, and the Q-1G allele were significantly higher in cases than in controls (*P *< 0.001, OR = 2.81, 95% CI = 2.19-3.61; *P *< 0.001, OR = 2.60, 95% CI = 2.06-3.30; *P *< 0.001, OR = 1.93, 95% CI = 1.50-2.50, respectively). Statistically significant differences in the distributions of the three alleles were observed between the two groups. The frequency of the S2C allele was slightly higher in the case group than in the control group (*P *= 0.031, OR = 1.31, 95% CI = 1.02-1.69). The details are shown in Table 5.

**Table 4:** ADAM33 genotype frequencies in cases and controls

		Frequency No (%)		
			
Genotype		CASE(n = 312)	CONTROL(n = 319)	*P*-value
V4	CC	200(64.10)	215(67.40)	0.3830
	CG	93(29.81)	88(27.59)	0.5373
	GG	19(6.09)	16(5.01)	0.5556
T+1	AA	9(2.88)	7(2.20)	0.5813
	AG	63(20.20)	64(20.06)	0.9676
	GG	240(76.92)	248(77.74)	0.8057
T2	AA	11(3.53)	60(18.81)	< 0.0001
	AG	100(32.05)	139(43.57)	0.0003
	GG	201(64.42)	120(37.62)	< 0.0001
T1	AA	83(26.60)	179(56.11)	< 0.0001
	AG	170(54.49)	122(38.24)	< 0.0001
	GG	59(18.91)	18(5.65)	< 0.0001
S2	CC	30(9.62)	23(7.21)	0.2761
	CG	121(38.78)	105(32.92)	0.1244
	GG	161(51.60)	191(59.87)	0.0365
S1	AA	171(54.81)	200(62.70)	0.0441
	AG	120(38.46)	99(31.03)	0.0501
	GG	21(6.73)	20(6.27)	0.8142
Q-1	AA	13(4.17)	40(12.54)	0.0002
	AG	100(32.05)	130(40.75)	0.0232
	GG	199(62.38)	149(46.71)	< 0.0001
F+1	CC	154(49.36)	159(49.84)	0.9032
	CT	128(41.03)	138(43.26)	0.5698
	TT	30(9.61)	22(6.90)	0.2143

**Table 5:** Association of ADAM33 alleles with COPD

		Minor allele frequency			
				
SNP	Allele	Case	Control	*P*-value	Odds ratio (95% CI)
V4	G:C	0.210	0.188	0.3310	1.15 (0.87-1.51)
T+1	G:A	0.130	0.122	0.6861	1.07 (0.77-1.49)
T2	G:A	0.196	0.406	< 0.0001	2.81 (2.19-3.61)
T1	A:G	0.462	0.248	< 0.0001	2.60 (2.06-3.30)
S2	G:C	0.290	0.237	0.0313	1.31 (1.02-1.69)
S1	A:G	0.260	0.218	0.0819	1.25 (0.97-1.63)
Q-1	G:A	0.202	0.329	< 0.0001	1.93 (1.50-2.50)
F+1	C:T	0.301	0.285	0.5321	1.08 (0.85-1.38)

### ADAM33 haplotype analysis

We constructed the haplotypes of cases and controls. Haplotypes with frequencies >2% were selected for analysis. There were nine haplotypes in all samples. The frequency of haplotype CGGGGAGC was significantly higher in cases than in controls (*P *= 0.0002). The frequencies of haplotypes CGGGGAGT, CGGGCAGC, and CGGGGGGC were also significantly higher in cases (*P *= 0.0001, 0.0005, and 0.0074, respectively). In contrast, the haplotype CGAAGAGC was more common in the control group than in the case group (*P *< 0.0001). The details are listed in Table 6.

**Table 6:** Association of ADAM33 haplotypes with COPD

		Frequencies		
			
Haplotype	Frequencies	Case	Control	*P *value of χ^2^-test
CGGAGAGC	0.122	0.125	0.199	0.7353
CGAAGAGC	0.064	0.032	0.096	< 0.0001
CGGGGAGC	0.053	0.077	0.030	0.0002
GGGAGAGC	0.034	0.035	0.034	0.8664
CGGAGAGT	0.034	0.034	0.033	0.908
CGGGGAGT	0.03	0.049	0.012	0.0001
CGGGCAGC	0.027	0.043	0.011	0.0005
CGGGGGGC	0.027	0.039	0.015	0.0074
CGGACAGC	0.023	0.029	0.018	0.1904

## Discussion

The present study is the first demonstration of an association between ADAM33 polymorphisms and COPD in an East Asian population. Using a case-control design, we investigated the relationship between human ADAM33 polymorphisms and COPD in northeast China. Our results showed that the T1, T2, S2, and Q-1 SNPs were significantly associated with COPD. As multiple SNPs may act in combination to increase the risk of COPD, haplotypes were constructed, and their frequencies were compared between the case and control groups. The haplotype data suggests that the CGGGGAGC, CGGGGAGT, CGGGCAGC, and CGGGGGGC haplotypes may be risk factors for COPD. In contrast, the CGAAGAGC haplotype may be a protective factor for the disease.

It is known that lung function decline is a risk factor for the development of COPD and cardiovascular disease [23]. Lee and coworkers demonstrated that ADAM33 protein levels in bronchoalveolar lavage fluid (BALF) are inversely correlated with predicted FEV1 values in patients with asthma [20]. Associations of polymorphisms in ADAM33 with FEV1 decline may therefore constitute a risk for the development of COPD as well. In previous studies, it has been shown that polymorphisms in the ADAM33 gene play a role not only in asthma susceptibility, but in its progression [16]. In a Dutch population, the SNPs S1, S2, and Q-1 were associated with FEV1 decline, and the SNPs S1, S2, F+1, and T2 were associated with the presence of COPD [21]. In another study of European children, SNPs F+1, M+1, T1, and T2 were associated with lower FEV1 [24]. These conflicting results could be due to ethnic differences between the Caucasian populations and the East Asian population in the present study. Another possibility is that there may be differences in the genetic background of COPD in Chinese, Caucasian, and Dutch populations. Therefore, further studies investigating the distributions of the ADAM33 gene variants in other populations is required.

In our study, three SNPs were associated with COPD. The T1 and T2 SNPs are located in the exon 19 (which includes an SH3 domain and a phosphorylation site) of the cytoplasmic tail, which may affect signaling. The SNP Q-1 is located in the intron immediately before exon 16, which contains an epidermal growth factor (EGF) domain [25]. EGF signaling is important in lung morphogenesis, and mice lacking the EGF receptor (EGFR) demonstrate abnormal branching and poor alveolarization. EGFR signaling regulates matrix metalloproteases, which mediate epithelial-mesenchymal interactions during lung morphogenesis [26]. ADAM33 is closely related to matrix metalloproteases, but may bind EGF directly. A disturbance in the EGF domain will likely affect regulation of ADAM33. Through alterative splicing, exon 16 can be spliced out, giving rise to the β-variant of ADAM33. This variant was found in 30% of ADAM33 mRNA transcripts in pulmonary fibroblasts [27]. Because the EGF domain is incomplete, it has been suggested that the β-variant prevents maturation of ADAM33 and may exert a dominant-negative effect on its protease activity [28]. The intronic Q-1 SNP may therefore influence the splicing of the β-variant [29] and disturb the maturation of ADAM33. Subsequent effects on protease activity may result in a defect in tissue repair after inflammation-induced damage. This may lead to progressive destruction of alveolar tissue and thereby enhance accelerated decline in lung function.

We have previously shown that polymorphisms in the ADAM33 gene are associated with adult allergic asthma and rhinitis in a Chinese Han population [30]. Interestingly, we now present evidence that polymorphisms in ADAM33 are associated with COPD in a Chinese Han population. In these studies, some common SNPs are associated with all three diseases, suggesting that they contribute to accelerated airway dysfunction in various disease progressions. However, some specific SNPs are only associated with one disease. This phenomenon is consistent with the pathogenetic mechanisms of the three diseases. On one hand, the pathogenetic mechanisms of asthma, allergic rhinitis, and COPD are different from each other, and each disease has its specific diagnosis. On the other hand, there may be some overlapping aspects in their pathogenetic mechanisms. At present, the mechanistic roles of the disease-associated SNPs have yet to be elucidated, especially in the context of the pathophysiology of asthma and COPD. Further research on ADAM33 should clearly involve functional studies to elucidate the roles of ADAM33 SNPs in lung function loss, asthma, and COPD.

## Conclusion

In summary, our results suggest an association between the ADAM33 gene polymorphisms and COPD in the northeastern Chinese Han population.

## Competing interests

The authors declare that they have no competing interests.

## Authors' contributions

XW participated in the molecular study and drafted the manuscript. LL performed the statistical analysis. JX and CJ participated in the statistical analysis and performed haplotype reconstruction. KH and XK recruited volunteers. XW participated in the design of the study. FI initiated the study and participated in its design and coordination. All authors contributed to data interpretation and manuscript revisions, and all approved the final manuscript.

## Pre-publication history

The pre-publication history for this paper can be accessed here:

http://www.biomedcentral.com/1471-2350/10/132/prepub
